# Gegen-Qinlian decoction alleviates anxiety-like behaviors in methamphetamine-withdrawn mice by regulating *Akkermansia* and metabolism in the colon

**DOI:** 10.1186/s13020-023-00794-w

**Published:** 2023-07-16

**Authors:** Xue Lu, Yu Fan, Yaqin Peng, Weichao Pan, Demin Du, Xing Xu, Nanqin Li, Teng He, Jiaxun Nie, Pengbo Shi, Feifei Ge, Dekang Liu, Yugen Chen, Xiaowei Guan

**Affiliations:** 1 Department of Human Anatomy and Histoembryology, Nanjing University of Chinese Medicine, Nanjing, 210023 China; 2 Affiliated Hospital of Nanjing University of Chinese Medicine, Nanjing, China

**Keywords:** Methamphetamine withdrawal anxiety, Gut metabolism and microenvironment, *Akkermansia* growth and metabolism, Gegen-Qinlian decoction

## Abstract

**Background:**

Anxiety is a prominent withdrawal symptom of methamphetamine (Meth) addiction. Recently, the gut microbiota has been regarded as a promising target for modulating anxiety. Gegen-Qinlian decoction (GQD) is a classical Traditional Chinese Medicine applied in interventions of various gut disorders by balancing the gut microbiome. We aim to investigate whether GQD could alleviate Meth withdrawal anxiety through balancing gut microbiota and gut microenvironment.

**Methods:**

Meth withdrawal anxiety models were established in mice. GQD were intragastric administrated into Meth-withdrawn mice and controls. Gut permeability and inflammatory status were examined in mice. Germ-free (GF) and antibiotics-treated (Abx) mice were used to evaluate the role of gut bacteria in withdrawal anxiety. Gut microbiota was profiled with 16s rRNA sequencing in feces. Metabolomics in colon tissue and in *Akkermansia* culture medium were performed.

**Results:**

Meth withdrawal enhanced anxiety-like behaviors in wild-type mice, and altered gut permeability, and inflammatory status, while GQD treatment during the withdrawal period efficiently alleviated anxiety-like behaviors and improved gut microenvironment. Next, we found Germ-free (GF) and antibiotics-treated (Abx) mice did not develop anxiety-like behaviors by Meth withdrawal, indicating the essential role of gut bacteria in Meth withdrawal induced anxiety. Then, it was observed that gut microbiota was greatly affected in Meth-withdrawn mice, especially the reduction in *Akkermansia*. GQD can rescue the gut microbiota and reverse *Akkermansia* abundance in Meth-withdrawn mice. Meanwhile, GQD can also restore the Meth-impaired *Akkermansia* growth in vitro. Further, GQD restored several common metabolite levels both in colon in vivo and in *Akkermansia *in vitro.

**Conclusions:**

We revealed a novel effect of GQD on Meth withdrawal anxiety and identified its pharmacological target axis as “*Akkermansia-Akkermansia* metabolites-gut metabolites-gut microenvironment”. Our findings indicated that targeting gut bacteria with TCM, such as GQD, might be a promising therapeutic strategy for addiction and related withdrawal symptoms.

## Background

Methamphetamine (Meth) is a highly addictive psychostimulant drug, and the high risk of relapse remains a big public health concern. Anxiety is a prominent withdrawal symptom of Meth addiction [1], which was generally believed a critical factor for driving relapse [2]. Currently, there were limited efficacious treatment options for Meth withdrawal-induced anxiety, partially due to the toxic side effects of anxiolytic medicine on the brain [3]. Thus, exploring the peripheral targets for intervening METH withdrawal-induced anxiety is essential for developing clinical strategies and improving patient care.

In the recent decade, gut microbiome was emerging as an important contributor to substance abuse and related psychiatric comorbidities [4], including anxiety and depression [5–8]. In alcohol and cocaine addicts, the gut microbiota was severely affected, which in turn exacerbated addiction and psychiatric comorbidity [9, 10]. Moreover, mice with gut microbiota depletion exhibited higher sensitivity to a low dose of cocaine [11]. While, rats with different gut microbial compositions had various sensitivities to Meth, and Meth exposure could also change the gut microbiota composition in turn [12]. Based on these studies, we assumed that gut microbiota may act as a periphery target for modulating Meth withdrawal anxiety. However, the key bacteria in the gut implicated in Meth withdrawal anxiety remains unknown.

Traditional Chinese Medicine (TCM) has been widely applied in the management of Meth abuse, especially for Meth withdrawal syndrome [13, 14]. Gegen-Qinlian decoction (GQD) is a classical TCM formula that is composed of four herbs including *Pueraria lobata, Scutellaria baicalensis, Coptis chinensis* and *licorice*, and has been proven to be effective in treating diarrhea, ulcerative colitis and type 2 diabetes in clinic [15, 16]. Traditionally, GQD exerted its therapeutic effects through modulating the gut microenvironment, such as homeostasis of inflammation, oxidation and neuropeptide release [17–19]. Recently, emerging evidence demonstrated that GQD can remodel the gut microbiome [20–22]. In addition, puerarin, a key active component in GQD, can protect neurons in dementia [23] and Parkinson’s Disease [24] in rodent models. As such, we hypothesized that GQD may be a potential therapeutic strategy for Meth withdrawal anxiety, probably through balancing the gut microbiota and gut microenvironment.

In the present study, models of long-term Meth withdrawal, Germ-free (GF) and antibiotics-treated exposure (ABx) were established in mice, and *Akkermansia* culture was performed in vitro. The link between anxiety and gut bacteria in Meth-withdrawn mice, as well as the potential pharmacological “gut bacteria-gut microenvironment” targets of GQD on Meth withdrawal anxiety were explored in vitro and in vivo.

## Materials and methods

### Mice breeding and drug administration

The animal breeding and all behavioral tests with mice were approved and supervised by the Guide for the Care and Use of Laboratory Animals at Nanjing University of Chinese Medicine (Approval N: NJUCM21-080). C57BL/6 wild type (WT) male mice with 8 weeks were used in our model and caged for at least 3 days for acclimation before Meth administration. All mice were kept at constant temperature (23 ± 2 ℃) and humidity (50 ± 5%) under a 12-h light/dark cycle (lights on at 8:00 AM) and were accessible for water and food. Besides, Germ Free (GF) mice were purchased from GemPharmatech and maintained in a sterile box. Antibiotics exposure mice (ABx) were generated by intragastric administrating (i.g.) mice with 100 mg/kg neomycin, 50 mg/kg vancomycin and 100 mg/kg metronidazole twice per day and free access to water containing 1 mg/ml ampicillin for 7 days.

Mice were subject to intraperitoneal injection (i.p.) with saline or 1 mg/kg Meth (dissolved in saline) consecutively for the indicated period, followed by w/o 0.2 ml GQD intragastric administration (i.g.) every two days for the indicated period. Then, mice were immediately subject to behavioral tests.

### GQD preparation

24 g *Puerariae lobatae radix*, 9 g *Coptidis rhizoma*, 9 g *Scutellariae radix* and 6 g *Glycyrrhizae radix* were soaked in cold 500 ml water for 30 min, then heated with strong fire to ebullition followed by gentle heat for 30 min. The filtrated were obtained as the first decoction. The filtered residue was added with 500 ml cold water, boiled and gentle heat for 30 min, then filtrated to be second decoction. Finally, the first and second decoctions were mixed, thoroughly filtered from herbs dregs, rotary evaporated, lyophilized and stored at 4 ℃ for further use.

### Behavioral testing

Before all behavioral tests, mice were habituated in the testing room for at least 2 h and all the tests were performed during the light cycle. All data were recorded and analyzed by TopScan H system (TopScan, USA).

Elevated plus maze test (EPM) and Open field test (OFT) were embraced in our research to reflect the anxiety level of mice. In EPM, mice were placed in a potentially dangerous apparatus, which consists of four intersect elevated arms (52 cm above the floor) with two 1 cm walled arms (open arms) and two 40 cm walled arms (closed arms). At the start of the assay, mice were placed at the center of the apparatus (cross point) with head to one of the open arms. Then the mice were allowed to explore freely in the apparatus for 5 min. The durations in open arms, the times of entry into open arms, and the total distance traveled in the apparatus were recorded. In OFT, mice were initially placed to the corner of one open box (50 × 50 × 50 cm) and allowed to explore freely for 5 min. the duration, the entry times into the center area (16 × 16 cm), and the total distance traveled in the apparatus were recorded. TopScan software was applied in recording and analyzing data in mice behavioral tests.

### 16s rRNA sequencing and analysis

Mice were sacrificed and fecal samples were collected from the colon into 2 ml tubes, immediately snapping frozen into liquid nitrogen. The samples were stored at − 80 ℃ for further analysis.

The absolute quantifications of 16s rRNA of gut microbial were performed by Shanghai Genesky Biotechnologies (China). Briefly, bacteria genomic DNA was extracted with FastDNA® SPIN Kit (MP Biomedicals, Santa Ana, CA, United States). The quality of genomic DNA was examined by agarose gel electrophoresis, and the concentration and purity were determined by Nanodrop 2000 and Qubit3.0 Spectrophotometer. Polymorphic spike-ins consisted of identical conserved regions to 16s rRNA and variable regions with randomized sequences (GC% ~ 40%) which acted as quantification labels. Then, proper proportions of spike-ins with gradient copy numbers were mixed with genomic DNA to amplify V3-V4 regions of the 16 rRNA and spike-ins using primers 341F (5′-CCTACGGGNGGCWGCAG-3′) and 805R (5′-GACTACHVGGGTATCTAATCC-3′), and finally sequenced using Illumina NovaSeq 6000 sequencer according to manufacturer’s instructions. The relative 16s rRNA sequencing of fecal bacteria gDNA adopted the same process without carrying out the spike-ins in amplification mixtures.

The initial processing of raw read sequences involved using QIIME2. The cutadapt plugin was utilized to remove adaptor and primer sequences. To ensure high quality data and identify amplicon sequence variants (ASVs), the DADA2 plugin was employed. The taxonomic assignments of ASV representative sequences were conducted using a pretrained Naive Bayes Classifier with a confidence threshold of 0.8. This classifier was trained on Greengenes ver. 13.8. Following this, spike-in sequences were identified, and reads were counted. A standard curve was generated for each sample based on the read counts vs. spike-in copy number, and the absolute copy number of each ASV in each sample was determined by using the corresponding ASV read counts. Since the spike-in sequence is not part of the sample flora, it must be eliminated from subsequent analysis.

### In vitro* Akkermansia* culture

*Akkermansia muciniphila* was a gift from Y Chen’s Lab in the Affiliated Hospital of Nanjing University of Chinese Medicine. *Akkermansia* was stored at − 80 ℃, thawed at 4℃ before use and subsequently inoculated into conditional medium (CM), cultured in an anaerobic workstation with 10% H_2_, 5% CO_2_, 85% N_2_ at 37 ℃. The CM preparation: 50–100 mg fresh mice feces were thoroughly washed with 1 ml PBS, and concentrated with 1000*g* centrifuge into a small volume of semisolid fluid, which subsequently aliquoted 500 μl with 200 μl LB (Luria–Bertani) medium per tube, added by indicated amount of Meth or GQD. The mix was then incubated in an anaerobic workstation for 4 h and the supernatant was collected by centrifuge to get Meth-CM and GQD-CM culture ready for use.

### Histology, immunofluorescence and western blotting

The tissues were obtained from sacrificed 4% PFA (paraformaldehyde) prefixed mice and furtherly fixed with 4% PFA for 4 h. Tissues underwent serious dehydration, cleared in xylene and embedded in paraffin. 5 µm sections were used for H&E staining and immunofluorescence assays, performed according to canonical procedures described before or the protocol provided by the manufacturers of primary antibodies. The following antibodies were used in immunofluorescence assays: Claudin-3 (Abcam, ab214487), ZO-1 (Abcam, ab190085). The light and fluorescence microscopy photos were analyzed by Leica LAS X software.

For western blotting assay, fresh tissues were incubated with RIPA lysis buffer containing proper amount of proteinase and phosphatase inhibitors in Ball milling machine for 1 min, followed by staying on ice for 30 min. Then, the supernatant of the lysate was harvested by centrifuge to get the protein extract. The protein concentrations were determined by BCA reagent. The protein were segregated by SDS-PAGE electrophoresis, transferred to PVDF membrane and incubated against the indicated primary antibody. The following antibodies were used: Claudin-3 (Abcam, ab214487), ZO-1 (Abcam, ab190085), TNF-α (Proteintech, 17590-1-AP), IL-1β (CST, 12703s), IL-6 (Proteintech, 66146-l-Ig), IL-10 (Proteintech, 20850-l-AP), β-actin (Proteintech, 66009-l-Ig). ImageJ was used for intensity quantifications of blotting assays.

### Untargeted metabolomics analysis

The feces sample were collected freshly from mice colons 40 min after behavioral tests, then dounced with liquid nitrogen and resuspended with prechilled 80% methanol. The mix was incubated on ice for 5 min and the clear supernatant were harvested by centrifuge twice at 15,000*g* at 4 ℃ for 20 min, and subsequently injected into LC–MS/MS system for analysis [25].

The CM culture was prepared as previously described, followed by adding saline (SV), 8 μg/ml Meth (MV), 50 μg/ml GQD (SG), or 8 μg/ml Meth and 50 μg/ml GQD (MG). After 24-h anaerobically *Akkermansia* cultured, the CM samples were collected for metabolomics analysis.

UHPLC-MS/MS analyses were conducted with Vanquish UHPLC system (ThermoFisher, Germany) coupled with an Orbitrap Q ExactiveTMHF-X mass spectrometer (Thermo Fisher, Germany) in Novogene (Beijing, China), according to manufacturer’s instructions.

The raw data files generated by UHPLC-MS/MS were processed using Compound Discoverer 3.1 (CD3.1, ThermoFisher) according to the optimized protocols by Novogene. Then, the accurate qualitative and relative quantitative results were obtained by normalizing peak intensities to the total spectral intensities using mzCloud (https://www.mzcloud.org/).

For processed data, Software R (R version R-3.4.3), Python (Python 2.7.6 version) and CentOS (CentOS release 6.6) were utilized for statistical analyses. Kyoto Encyclopedia of Genes and Genomes (KEGG) database (https://www.genome.jp/kegg/pathway.html), HMDB database (https://hmdb.ca/metabolites) and LIPIDMaps database (http://www.lipidmaps.org/) were used for metabolites classification annotation. Principal components analysis (PCA) and Partial least squares discriminant analysis (PLS-DA) was performed at metaX. Volcano plots were used to filter metabolites of interest by ggplot2 in R language. Pheatmap package in R language was used for clustering heat maps and differential metabolites correlation analysis.

### Data and statistical analyses

All data were presented as the mean ± SD. Statistical analyses were performed by GraphPad Prism 8. The behavioral data in GF and ABx mice were analyzed by unpaired *t*-tests. The data of *Akkermansia* growth in vitro were analyzed by one-way ANOVA followed by multi-compared tests at each time point. Other data were analyzed by two-way ANOVA followed by multi-compared tests. Statistical significance was set as *P* < 0.05.

## Results

### GQD alleviates anxiety-like behaviors and improves gut microenvironment in Meth-withdrawn mice

**Fig. 1:**
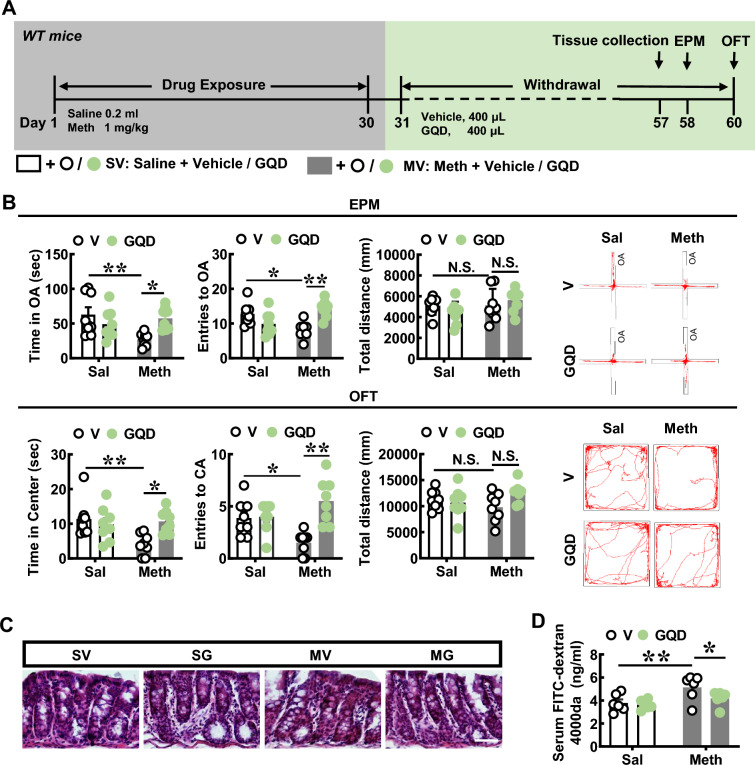

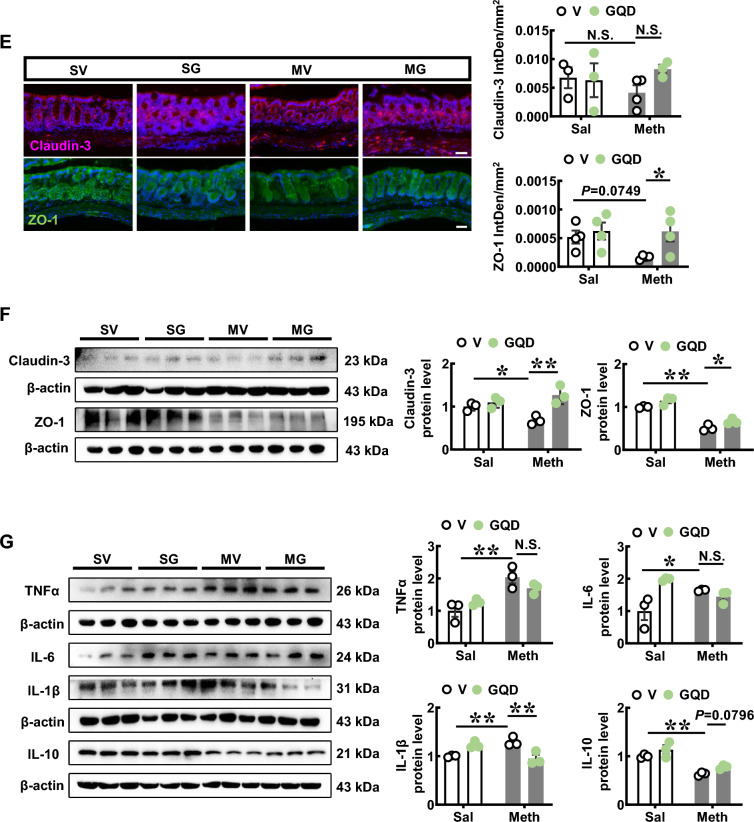
GQD alleviates anxiety-like behaviors and improved gut microenvironment in Meth-withdrawn mice. **A** The experimental timeline settings in wild-type (WT) mice. **B** Anxiety-like behavior tests, including elevated plus maze test (EPM) and open field test (OFT). Pictures to the right show the representative traces of the corresponding behavioral tests. **C** The H&E staining pictures of colon mucosa. Scale bar: 50 μm. **D** The colon permeability test with FITC-Dextran 4000da detection. **E** The staining densities of Claudin-3 or ZO-1 in colon mucosa. Scale bar: 50 μm. **F** Western blotting of Claudin-3 or ZO-1 protein levels in colon. **G** Western blotting of several pro-inflammatory factors, including TNFα, IL-6 and IL-1β, and the anti-inflammatory factor IL-10 in colon. OA: open arms of EMP. CA: central area of OFT. Sal: saline withdrawal mice. Meth: Meth-withdrawn mice. V: vehicle-treated mice. GQD: GQD-treated mice. N.S., *P* > 0.05; *, *P* < 0.05; **, *P* < 0.01 versus the indicated controls

To test the role of GQD in the modulation of anxiety-like behaviors in Meth-withdrawn mice, mice were treated with GQD during the withdrawal period (Fig. 1A). When compared with saline withdrawal mice (Sal), Meth-withdrawn mice (Meth) spent a shorter time (n = 16, *P* = 0.0094, Fig. 1B) and fewer entries (n = 16, *P* = 0.0139, Fig. 1B) into open arms of EPM, and shorter time (n = 16, *P* = 0.0094, Fig. 1B) and less entries into central area (n = 16, *P* = 0.0338, Fig. 1B) of OFT. Compared with vehicle treatment (V), GQD treatment increased the spent time (n = 16, *P* = 0.0323, Fig. 1B) and entries into open arms (n = 16, *P* = 0.0009, Fig. 1B) of EPM, and increased the time in central area (n = 16, *P* = 0.0215, Fig. 1B) and entries into field center (n = 16, *P* = 0.0002, Fig. 1B) of OFT in Meth-withdrawn mice. Both Meth withdrawal and GQD treatment did not influence the locomotive behaviors in mice of four groups, as shown by similar total distance traveled in EPM (n = 16, *P* > 0.05, Sal vs. Meth, V-Meth vs. GQD-Meth) and OFT (n = 16, *P* > 0.05, Sal vs. Meth, V-Meth vs. GQD-Meth) apparatus (Fig. 1B). These results indicated that GQD treatment effectively mitigated Meth withdrawal-induced anxiety.

Given that GQD can modulate the gut microenvironment, we examined the gut permeability and inflammatory status in Meth-withdrawn mice. As shown by colon H&E staining (Fig. 1C), both Meth withdrawal and/or GQD did not change the histological structure of colon. By FITC-Dextran detection in vivo, the gut permeability was increased in Meth-withdrawn mice (n = 12, *P* = 0.0066 *vs* vehicle-treated saline withdrawal mice), while GQD addition restore the permeability to a lower level (n = 12, *P* = 0.0462 *vs* vehicle-treated Meth-withdrawn mice, Fig. 1D). The tight junction protein levels of Claudin-3 and ZO-1 were assessed in colon. We found GQD treatment did not alter Claudin-3 densities of Meth-withdrawn mice (n = 6, *P* = 0.1346 *vs* vehicle-treated Meth-withdrawn mice, respectively), while GQD greatly restored the ZO-1 density of staining (n = 8, *P* = 0.0286) that was downregulated, though not significantly, by Meth withdrawal (n = 8, *P* = 0.0749 *vs* vehicle-treated saline withdrawal mice, Fig. 1E). Besides, Western blotting showed that the Claudin-3 (n = 6, *P* = 0.0239 vs vehicle-treated saline withdrawal mice) and ZO-1 levels (n = 6,* P* < 0.0001 *vs* vehicle-treated saline withdrawal mice) were downregulated in Meth-withdrawn mice, which were rescued by GQD treatment (n = 6, *P* = 0.0009 and *P* = 0.0297 vs vehicle-treated Meth-withdrawn mice, respectively, Fig. 1F). Together, these results indicated that Meth can induce the loss of tight junctions of colon epithelial cells while GQD can restore the tight junctions to some extent.

To assess the inflammatory status of colon, the protein levels of several pro-inflammatory factors, including TNFα, IL-6 and IL-1β, and the anti-inflammatory factor IL-10 were examined in colon. As shown in Fig. 1G, the protein levels of TNFα (n = 6, *P* = 0.0009 vs vehicle-treated saline withdrawal mice), IL6 (n = 6, *P* = 0.0174 vs vehicle-treated saline withdrawal mice), IL-1β (n = 6, *P* = 0.0039 vs vehicle-treated saline withdrawal mice) were elevated and IL-10 (n = 6, *P* = 0.0009 vs vehicle-treated saline withdrawal mice) was downregulated by Meth treatment, which were reversed by GQD (n = 6, *P* = 0.0016 for IL-1β, and n = 6, *P* = 0.0796 for IL-10 vs vehicle-treated Meth-withdrawn mice, respectively) except IL6 (n = 6, *P* = 0.3867 vs vehicle-treated Meth-withdrawn mice) and TNFα (n = 6, *P* = 0.1296 vs vehicle-treated Meth-withdrawn mice). These results indicated that GQD could rescue the aberrant inflammation response of colon by Meth withdrawal. Taking together, above results implied that GQD may regulate anxiety-like behaviors via modulating gut microenvironment status in Meth-withdrawn mice.

### GQD improves gut microbiota and the abundance of *Akkermansia* in Meth-withdrawn mice as well as in the culture in vitro

**Fig. 2:**
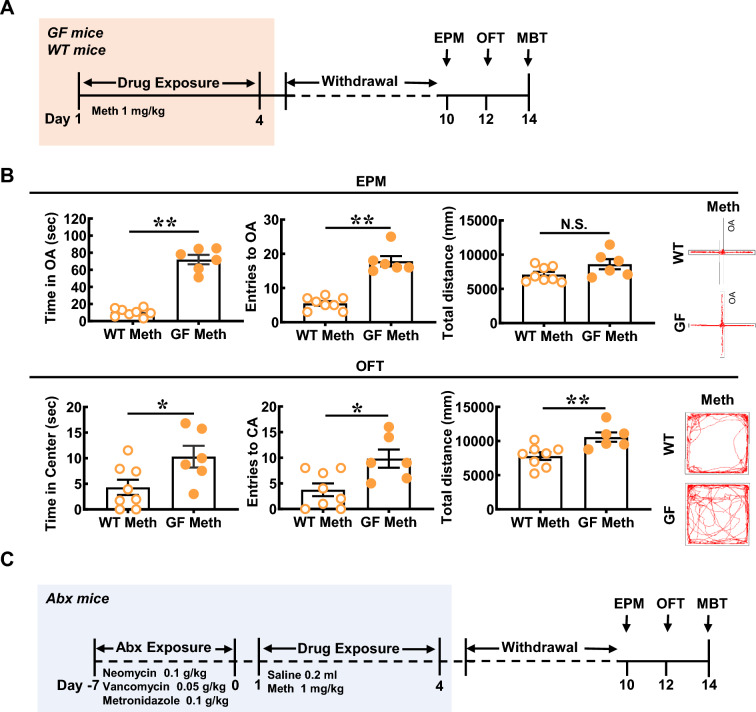

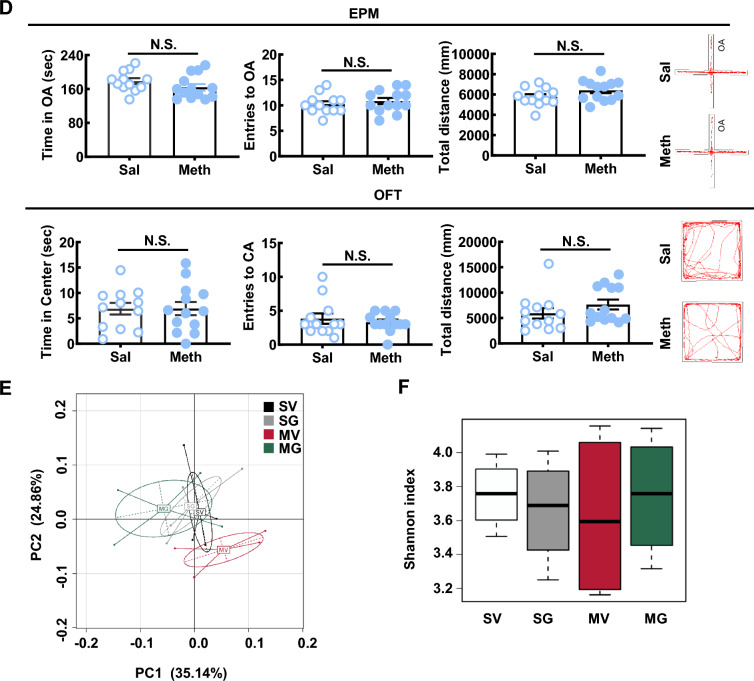

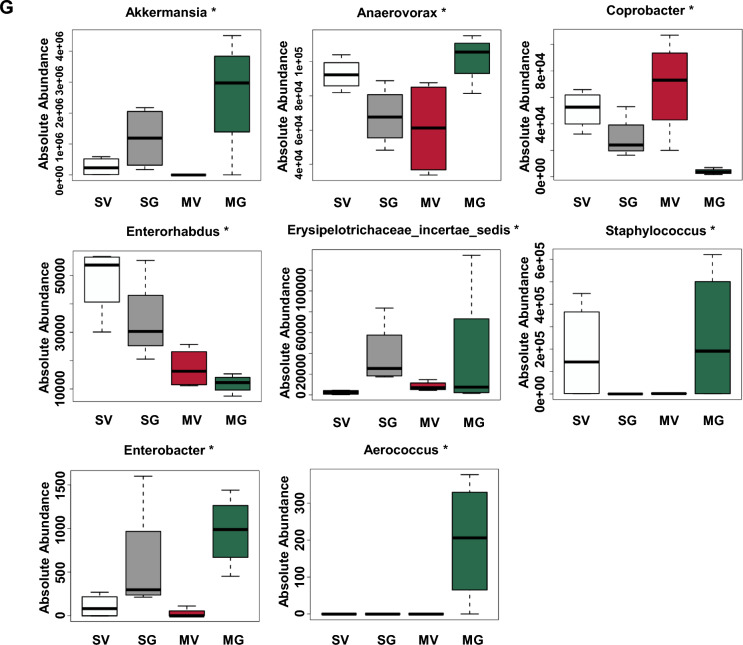

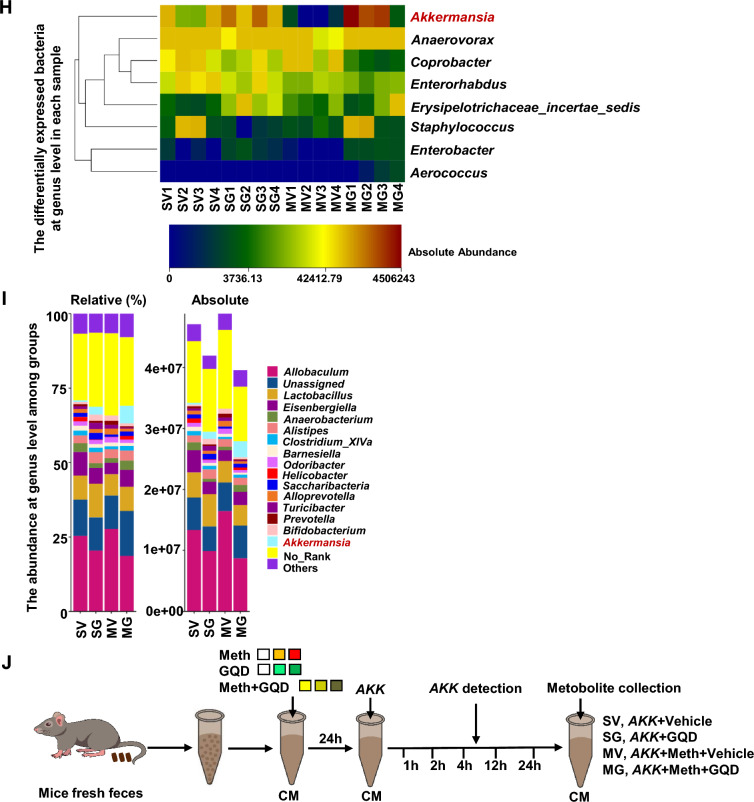

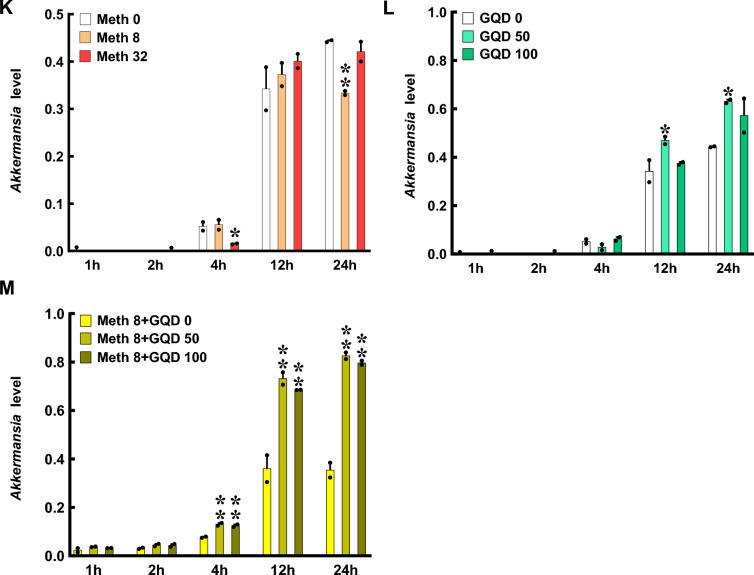
GQD improves gut microbiota and the abundance of *Akkermansia* in Meth-withdrawn mice as well as in the culture in vitro. **A** The experimental timeline settings in WT and Germ-free (GF) mice. **B** Anxiety-like behaviors tests, including EPM and OFT. Pictures to the right show the representative traces of the corresponding behavioral tests. **C** The experimental timeline settings in antibiotics exposure mice (ABx) mice. **D** Anxiety-like behaviors tests in ABx, including EPM and OFT. Pictures to the right show the representative traces of the corresponding behavioral tests. **E** Principal Coordinate Analysis (PCoA) among groups. **F** Shannon index the significant changed gut microbiota among groups. **G** Box graphs of significantly changed absolute abundances of eight bacteria at genus level among groups. **H** Heatmaps of bacteria flora with significant alternations in absolute abundance across SV, MV, SG and MG groups at genus level. **I** The abundances of all detected gut bacteria at genus level are illustrated by relative quantification (left) and absolute quantification (right). **J** The experimental setting of *Akkermansia *in vitro culture. **K**, **L** The growth profiles of *Akkermansia* with increasing concentrations (μg/ml) of Meth or GQD in culture medium. **M** The growth profiles of *Akkermansia* in 8 μg/ml Meth culture medium with increasing concentrations (μg/ml) of GQD. OA, open arms of EMP. CA, central area of OFT. Sal, saline withdrawal mice. Meth, Meth-withdrawn mice. V: vehicle-treated mice. GQD, GQD-treated mice. SV: vehicle treatment in saline withdrawal mice. MV: vehicle treatment in Meth-withdrawn mice. SG: GQD treatment in saline withdrawal mice. MG: GQD treatment in Meth-withdrawn mice. N.S., *P* > 0.05; *, *P* < 0.05; **, *P* < 0.01 versus the indicated or 0 concentration controls

To test the role of gut microbial in Meth withdrawal-induced anxiety, mice models of GF (GF, Fig. 2A) and ABx were used. When compared with Meth-withdrawn wild type (WT), Meth-withdrawn GF mice spent a longer time in open arms (n = 14, t = 12.12, *P* < 0.0001, Fig. 2B) and more entries into open arms (n = 14, t = 8.301, *P* < 0.0001, Fig. 2B) of EPM, and longer time in central area (n = 14, t = 2.389, *P* = 0.0342, Fig. 2B), more entries into field center (n = 14, t = 2.906, *P* = 0.0132, Fig. 2B) and longer traveled distance (n = 14, t = 3.149, *P* = 0.0084, Fig. 2B) in OFT, but similar distance traveled in EMP (n = 14, t = 1.944, *P* > 0.05, Fig. 2B), indicating GF mice are resistant to Meth withdrawal-induced anxiety with no locomotive abnormality. Likewise, there were no significant differences in the above spent time (EPM: n = 25, t = 1.399, *P* > 0.05; OFT: n = 25, t = 0.0134, *P* > 0.05), entries (EPM: n = 25, t = 0.7217, *P* > 0.05; OFT: n = 25, t = 0.6283, *P* > 0.05) and total distance (EPM: n = 25, t = 1.683, *P* > 0.05; OFT: n = 25, t = 1.217, *P* > 0.05) in both EPM and OFT between ABx Meth-withdrawn mice and ABx Sal-withdrawn mice (Fig. 2C, D). These results indicated that gut microbial participated in the ameliorating anxiety-like behaviors in Meth-withdrawn mice.

Then, 16s rRNA sequencing was conducted to analyze the compositions of gut microbial in mice of vehicle-treated or GQD-treated saline group (SV and SG) and Meth-withdrawn group (MV and MG). PCoA showed that the gut microbiota in flora samples of MV was obviously separated from the other 3 groups, and that of MG were closely clustered with SV and SG (Fig. 2E), indicating that Meth withdrawal could alter the composition of gut microbiota which could be restored by GQD treatment. Correspondingly, the Shannon index of the gut microbiota in MV was lower than that in SV, while GQD treatment increased the value in Meth withdrawal gut almost reaching the normal level (SV), indicating that Meth withdrawal decreased but GQD treatment could restore the gut microbial diversity (Fig. 2F). At genus levels, the absolute abundances level of eight floras were significantly altered by Meth withdrawal but restored by GQD treatment, including *Akkermansia*, *Anaerovorax*, *Coprobacter*, *Enterorhabdus*, *Erysipelotrichaceae*, *Staphylococcus*, *Enterobacter* and *Aerococcus* (Fig. 2G). Of the eight bacteria floras altered at genus leve, both the relative and absolute abundance of *Akkermansia* were among the tops, indicating its potential role as the targets of Meth and GQD (Fig. 2H, I). These results indicated that GQD might alleviate Meth withdrawal-altered composition and diversity of gut microbiota, especially that of *Akkermansia*.

In order to confirm the regulatory roles of Meth or GQD on *Akkermansia* growth, *Akkermansia* was cultured in vitro (Fig. 2J)*.* Here, to mimic the microbial community dynamics and relevant micro-environment within the mice colon, which benefit for the combinational analysis with in vivo metabolism data, we add naïve mice feces into the culture medium to perform Akkermansia growth in vitro. As shown in Fig. 2K, Meth at a concentration of 32 μg/ml significantly attenuated *Akkermansia* growth after 4-h culture (n = 4, *P* = 0.0468 vs Meth at concentration of 0), but returned to normal levels at 12-h (n = 4, *P* = 0.2752 vs Meth at concentration of 0) and 24-h culture (n = 4, *P* = 0.3002 vs Meth at concentration of 0). While, 8 μg/ml concentration of Meth could decrease *Akkermansia* growth after 24-h culture (n = 4, *P* = 0.0084 vs Meth at concentration of 0), indicating a long-term suppressing effect of 8 μg/ml dose of Meth in *Akkermansia* growth, mimicking gut bacteria in vitro. As shown in Fig. 2L, 50 μg/ml of GQD promoted, but 100 μg/ml of GQD did not affect the growth of *Akkermansia *in vitro at 12-h (50 μg/ml: n = 4, *P* = 0.0484 vs GQD at concentration of 0; 100 μg/ml: n = 4, *P* = 0.4759 vs GQD at concentration of 0) and 24-h culture (50 μg/ml: n = 4, *P* = 0.0476 vs GQD at concentration of 0; 100 μg/ml: n = 4, *P* = 0.1114 vs GQD at concentration of 0)*.* With 8 μg/ml of Meth, both 50 μg/ml and 100 μg/ml of GQD significantly promoted the growth of *Akkermansia* at 4-h (50 μg/ml: n = 4, *P* = 0.0037 vs 8 μg/ml of Meth + GQD at concentration of 0; 100 μg/ml: n = 4, *P* = 0.0051 vs 8 μg/ml of Meth + GQD at concentration of 0), 12-h (50 μg/ml: n = 4, *P* = 0.0050 vs 8 μg/ml of Meth + GQD at concentration of 0; 100 μg/ml: n = 4, *P* = 0.0073 vs 8 μg/ml of Meth + GQD at concentration of 0) and 24-h culture (50 μg/ml: n = 4, *P* = 0.0005 vs 8 μg/ml of Meth + GQD at concentration of 0; 100 μg/ml: n = 4, *P* = 0.0006 vs 8 μg/ml of Meth + GQD at concentration of 0, Fig. 2M). These results indicated that GQD can invert the inhibitory effect of Meth on *Akkermansia* growth in vitro.

### GQD ameliorates altered gut metabolism of Meth exposure mice

**Fig. 3:**
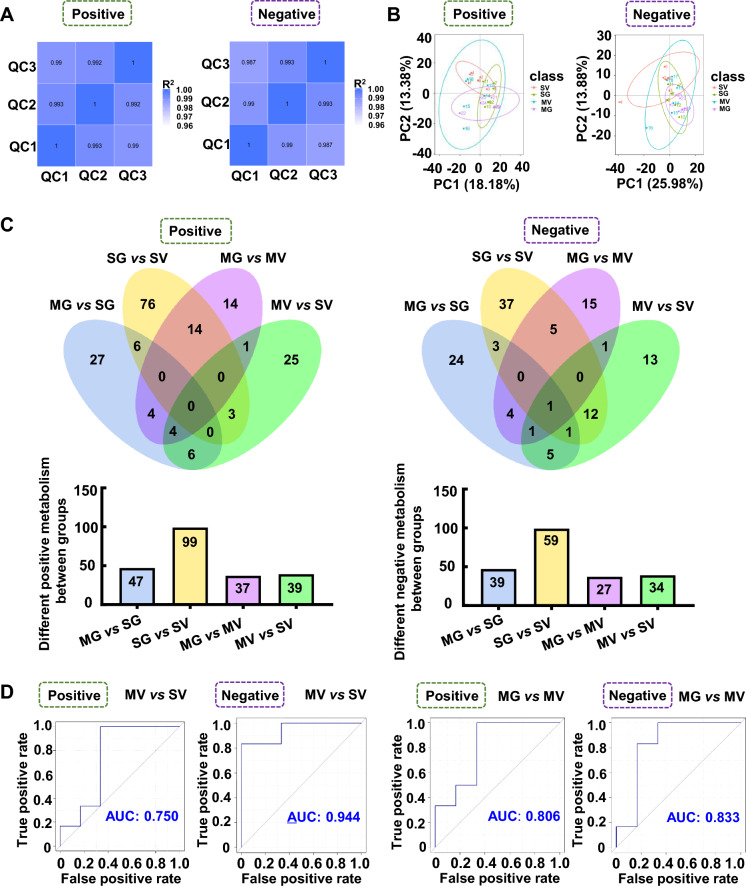

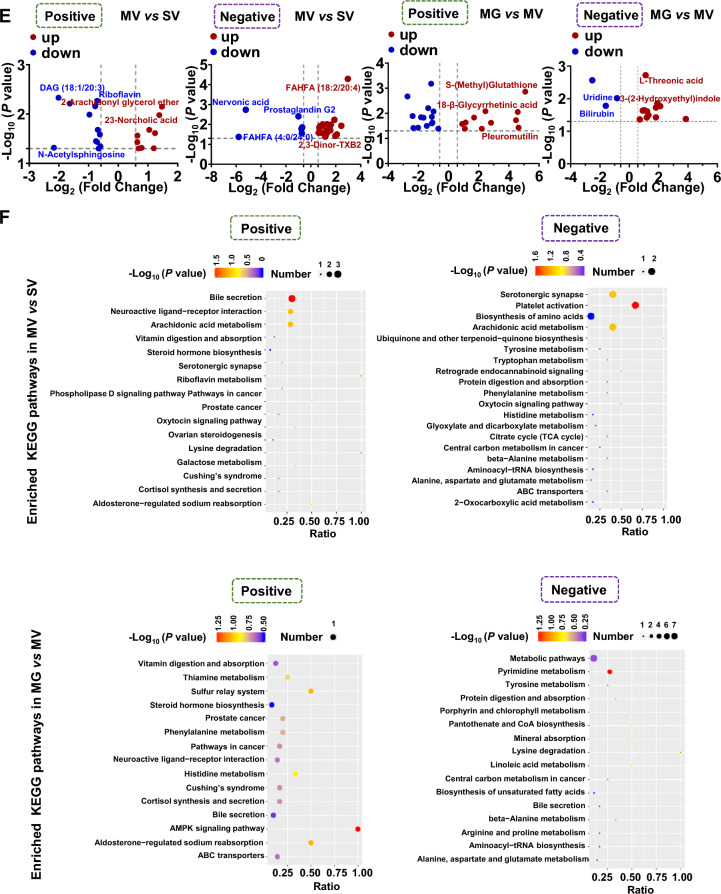

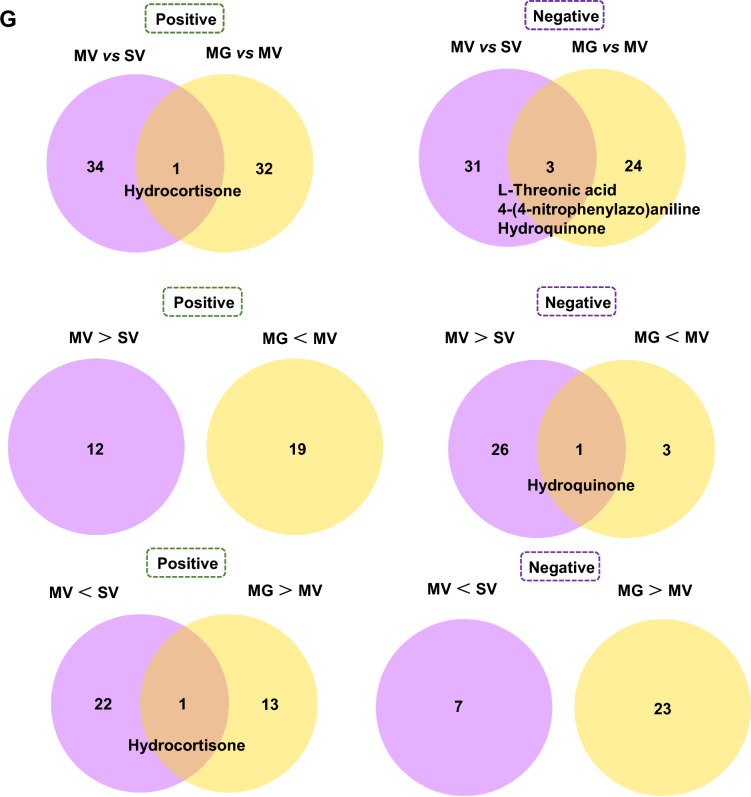
GQD improves Meth-altered mice colon metabolism in vivo. **A** PCA among groups. **B** PCoA among groups. **C** The Venn (upper) and column (lower) diagrams of different metabolites in colon among indicated groups. **D** The ROC curves and AUC of the screened-out differential metabolites of MV vs SV and MG vs MV. **E** Volcano graph of changed gut bacteria in MV vs SV and MG vs MV. **F** The top 10–20 enriched KEGG pathways of different metabolites in colon between SV and MV groups as well as MV and MG groups. **G** The comparison of changed *Akkermansia* metabolism in groups of MV vs SV and MG vs MV. SV, vehicle treatment in saline withdrawal mice. MV, vehicle treatment in Meth-withdrawn mice. SG, GQD treatment in saline withdrawal mice. MG, GQD treatment in Meth-withdrawn mice

To screen potential metabolic targets by GQD that might be linked with Meth withdrawal anxiety, the untargeted metabolomics profiling on gut metabolites was performed in the colon. The quality of collected samples was controlled by PCA (r > 0.99, Fig. 3A) and PCoA (Fig. 3B). Then, 99 and 59, 39 and 34, 37 and 27, 47 and 39 positive and negative differential metabolites were identified in SG vs SV, MV vs SV, MG vs MV and MG vs SG group, respectively (Fig. 3C). The value of AUC represented the high reliability of screened-out differential metabolites of MV vs SV and MG vs MV groups (Fig. 3D). The differential metabolites were enriched with KEGG bubble chart (Fig. 3E, F). Among them, nine enriched positive and six negative KEGG pathways of colon metabolites were both changed in groups of MV vs SV and MG vs MV (Table 1). Notably, among the differential metabolites, Hydroquinone was upregulated in MV compared to SV but downregulated with GQD supplementary in MG, and Hydrocortisone was downregulated in MV compared to SV but upregulated with GQD supplementary in MG (Fig. 3G).

**Table 1:** Common enriched KEGG pathways of gut metabolites in groups of MV vs SV and MG vs MV

	KEGG	MV vs SV	MG vs MV
Pos	Bile secretion	Hydrocortisone, Lithocholic acid; Prostaglandin F2α	Hydrocortisone
	Neuroactive ligand-receptor interaction	Hydrocortisone, Prostaglandin F2α	Hydrocortisone
	Aldosterone-regulated sodium reabsorption	Hydrocortisone	Hydrocortisone
	Prostate cancer	Hydrocortisone	Hydrocortisone
	Cortisol synthesis and secretion	Hydrocortisone	Hydrocortisone
	Cushing's syndrome	Hydrocortisone	Hydrocortisone
	Pathways in cancer	Hydrocortisone	Hydrocortisone
	Vitamin digestion and absorption	Riboflavin	Thiamine
	Steroid hormone biosynthesis	Hydrocortisone	Hydrocortisone
Neg	beta-Alanine metabolism	l-Histidine	Uracil
	Protein digestion and absorption	l-Histidine	l-Asparagine
	Tyrosine metabolism	Hydroquinone	Hydroquinone
	Central carbon metabolism in cancer	l-Histidine	l-Asparagine
	Aminoacyl-tRNA biosynthesis	l-Histidine	l-Asparagine
	Alanine, aspartate and glutamate metabolism	Citric acid	l-Asparagine

**Fig. 4:**
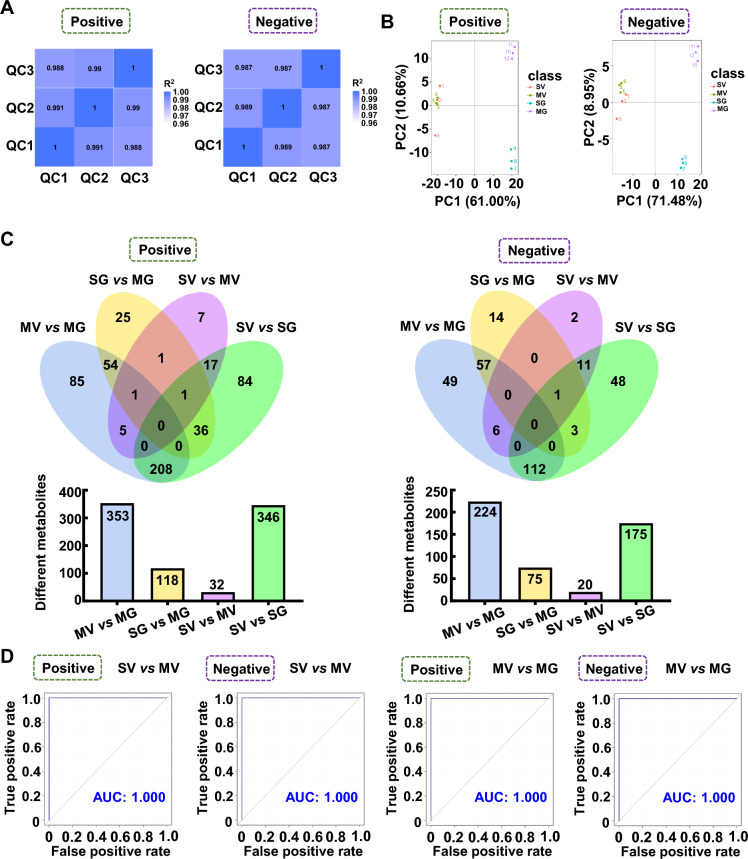

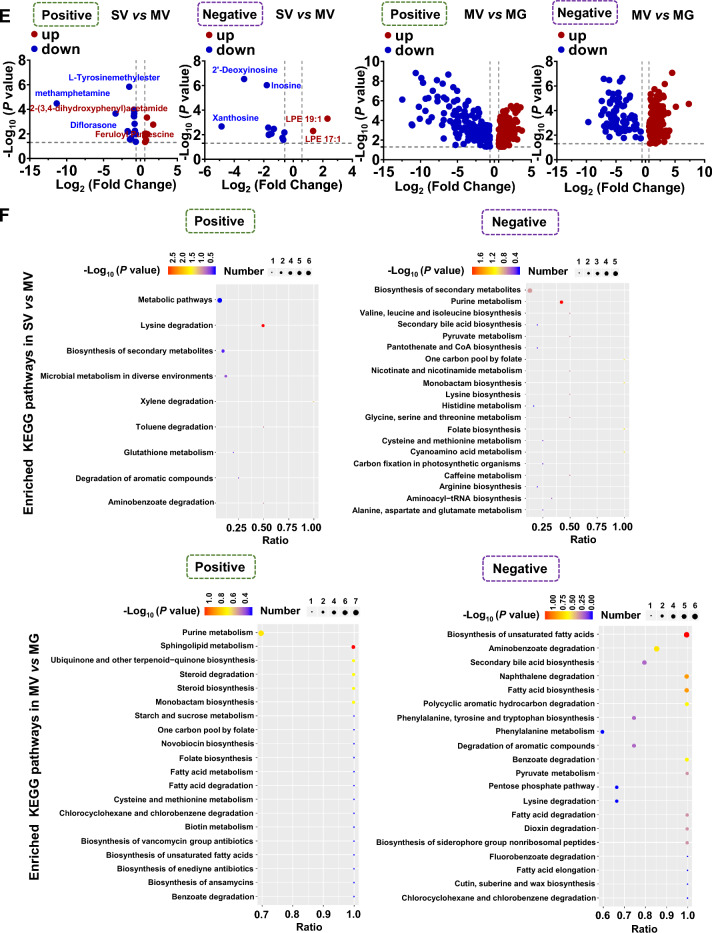

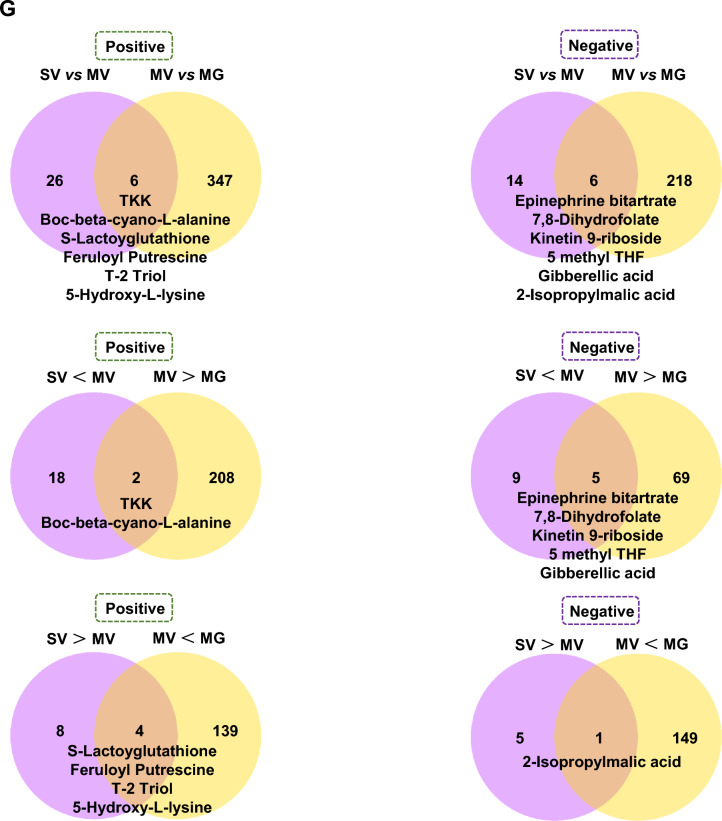
GQD restores Meth-impaired metabolism of *Akkermansia *in vitro. **A** Pearson correlation analysis (PCA) among groups. **B** PCoA among groups. **C** The Venn (upper) and column (lower) diagrams of different metabolites in *Akkermansia* culture medium among indicated groups. **D** The Receiver Operating Characteristic (ROC) curves and Area Under ROC Curve (AUC) of the screened-out differential metabolites of SV vs MV and MV vs MG. **E** Volcano graph of changed gut bacteria in SV vs MV and MV vs MG. **F** The top 10–20 enriched KEGG pathways of different metabolites in *Akkermansia* culture medium between SV and MV as well as MV and MG groups. **G** The comparison of changed *Akkermansia* metabolism in groups of SV vs MV and MV vs MG. SV, saline treatment in vehicle addition of *Akkermansia* culture medium. MV, Meth treatment in vehicle addition of *Akkermansia* culture medium. SG, saline treatment in GQD addition of *Akkermansia* culture medium. MG, Meth treatment in GQD addition of *Akkermansia* culture medium

Then, to target the potential metabolic molecules of *Akkermansia* that regulated gut microenvironment and Meth withdrawal anxiety, untargeted metabolomics profiling on the *Akkermansia* culture medium with saline (SV), GQD (SG), 8 μg/ml Meth addition (MV), and GQD & Meth addition (MG) were also performed. As shown by Pearson correlation analysis (PCA) and PCoA, the quality of collected samples met the predefined criteria (r > 0.99, Fig. 4A) and was separated clearly among groups (Fig. 4B). Overall, 346 and 175, 32 and 20, 353 and 224, 118 and 75 positive and negative differential metabolites were identified in SV vs SG, SV vs MV, MV vs MG and SG vs MG groups, respectively (Fig. 4C). The Receiver Operating Characteristic (ROC) curves and Area Under ROC Curve (AUC) illustrated the excellent separations and reliabilities of the screened-out differential metabolites of SV vs MV and MV vs MG (Fig. 4D), which were plotted by Volcano graph (Fig. 4E) and subject to KEGG database annotation. The top enriched KEGG pathways of different *Akkermansia* metabolites in groups of SV vs MV and MV vs MG were illustrated in Fig. 4F. Among them, two enriched KEGG pathways of *Akkermansia* metabolites, including pyruvate metabolism and secondary bile acid biosynthesis, were both changed in groups of SV vs MV and MV vs MG (Table 2). Notably, we found 7 metabolites that are upregulated in MV compared to SV but downregulated with GQD supplementary in MG, and 5 metabolites that are downregulated in MV compared to SV but upregulated with GQD supplementary in MG (Fig. 4G). Most importantly, GQD downregulated (6E)-7-(2H-1,3-benzodioxol-5-yl)-1-(piperidin-1-yl)hept-6-en-1-one and palmitoyl ethanolamide, while upregulated s-(methyl)glutathione, 18-β-glycyrrhetinic acid, berberine and dimethyl 4-hydroxyisophthalate simultaneously in *Akkermansia* culture medium and colon when exposed to Meth (Table 3).

**Table 2:** Common enriched KEGG pathways of *Akkermansia* metabolites in groups of SV vs MV and MV vs MG

Mode	KEGG	SV vs MV	MV vs MG
Neg	Pyruvate metabolism	2-Isopropylmalic acid	2-Isopropylmalic acid l-Malate
	Secondary bile acid biosynthesis	Cholic acid	Taurocholic acid, Glycocholic acid, Lithocholic Acid Deoxycholic acid

**Table 3:** Common down- or upregulated metabolites in MV vs MG groups in *Akkermansia* culture as well as in colon

Mode	MV > MG	MV < MG
Pos	(6E)-7-(2H-1,3-benzodioxol-5-yl)-1-(piperidin-1-yl)hept-6-en-1-one	S-(Methyl)Glutathione
	Palmitoyl ethanolamide	18-β-Glycyrrhetinic acid
		Berberine
Neg		Dimethyl 4-Hydroxyisophthalate

## Discussion

In recent decades, the gut microbiota was recognized as a regulator for not only gut function but also brain physiology [4]. As a result, many studies have been concerning gut microbiota as a valuable target for management of psychiatric disorders [26–29]. The Meth withdrawal symptoms, including anxiety, depression and cognition impairment, are severe neurobehavioral disorders that lack efficacious intervention approaches. Combining previous reports [30–32] with our findings that Meth abuse or abrupt cessation greatly altered the composition of gut microbial, we proposed that gut microbiota may be potential therapeutic strategies to regulate Meth withdrawal symptoms. To this end, here we found that the absence of gut microbial (GF mice) greatly affected the Meth withdrawal anxiety, corresponding well to one previous report [33]. Worthy to mention, we not only utilized GF mice but also antibiotics-treated (ABx) mice to ‘double check’ our result and consolidate our conclusion that gut microbial play critical roles in Meth withdrawal anxiety. In other reports, GF mice exhibit similar, more or fewer anxiety behaviors compared to WT mice in diverse conditions [34–37]. The discrepancy among these studies may reflect the unique mechanism underlying Meth withdrawal anxiety as well as the influence of different genetic backgrounds of mice.

GQD, a classic Chinese Medicine Decoction, has been applied clinically in treatment of intestinal diseases, like diarrhea and enteritis. As expected, GQD treatment can significantly attenuate anxiety level in Meth-withdrawn mice. Although a few reports were concerning the roles of TCM (mostly monomer) in antagonizing Meth associated neuro-damages [38–42], this study, to our knowledge, firstly reported that GQD mitigated the Meth withdrawal neurobehavioral abnormalities. Recently, by improving the compositions of gut microbial, GQD shows a desirable therapeutic effect on diseases like diabetes, cancers, ulcerative colitis, obesity and hypertension [18–22]. Combining the above results using GF and ABx mice, we assume that GQD may interfere the Meth withdrawal anxiety probably via modulating gut microbiota.

To find out potential bacteria targets of GQD in Meth-withdrawn anxiety, we conducted the 16s rRNA sequencing of gut microbial in mice. We found that Meth withdrawal greatly altered the compositions of gut microbial and increased microbial community diversity. These results were consistent with one recent research using human Meth withdrawal samples and another study with rats [31, 43]. In many brain disorders, including chronic stress, anxiety or depression, neurodegenerative diseases, schizophrenia and autism spectrum disorders, the microbiota diversity was found to be downregulated in the gut [44–47], supposing that the Meth withdrawal has different toxic effects on gut microbiota from other psychiatric disorders. In this research, we found that GQD intervention effectively rescued the Meth withdrawal-impaired microbiota community diversity to some extent, and simultaneously mitigated the Meth withdrawal anxiety. Of note, the microbiota diversity of saline-withdrawn mice was also significantly increased with GQD treatment. Few studies investigated the GQD effect on gut microbiota in naive mice or healthy humans, but at least our results indicated that the potential ‘side-effects’ of GQD should be noticed, even though the GQD-treated saline mice did not show any changes in anxiety-like behaviors. In analyzing the gut microbiota compositions, we found the abundances of eight bacteria altered at genus level across SV, SG, MV and MG groups. Among these bacteria, we assumed *Akkermansia* may act as the key bacteria in regulating Meth withdrawal anxiety with GQD administration, for the following reasons: (1) In the present study, Kruskal–Wallis test was utilized to take all four groups into comparison, thereby the screened-out candidate bacteria must be influenced significantly by Meth and GQD simultaneously; (2) Of eight bacteria altered at genus level, only *Akkermansia* and *Anaerovorax* belong to probiotics, which can improve the host metabolic modulations, immune responses as well as the efficacy of various cancers’ therapy approaches [48]. However, the variation trend of *Anaerovorax* abundances is inconclusive across SV, SG, MV and MG; (3) At genus level, *Akkermansia* was detected as one of the most abundant bacteria in mice faeces here; (4) Many studies show the positive relationship between *Akkermansia* abundance and anxiety [49–53]; (5) In vitro bacteria culture proved that GQD can independently promote the growth rate of *Akkermansia* and invert the inhibitory effect of Meth on *Akkermansia* growth.

To determine the specific molecules that may be involved in Meth withdrawal anxiety and GQD treatment, the untargeted metabolomics profiling was performed with the mice colon as well as *in Akkermansia* culture. In analyzing the colon metabolites, we found GQD can restore hydroquinone and hydrocortisone levels that are significantly altered by Meth administration. Meanwhile, hydrocortisone was widely involved in common enriched KEGG pathways of SV vs MV and MV vs MG. Unfortunately, Hydrocortisone did not alter with the same pattern in *Akkermansia* CM after Meth or GQD addition in vitro. Both in colon and in *Akkermansia* CM culture, we discovered two downregulated ((6E)-7-(2H-1,3-benzodioxol-5-yl)-1-(piperidin-1-yl) hept-6-en-1-one, palmitoyl ethanolamide), and three upregulated (S-(Methyl)Glutathione, 18-β-glycyrrhetinic acid, berberine, dimethyl 4-Hydroxyisophthalate) metabolites, when comparing the metabolites in MV vs MG*.* When screening out the commonly changed metabolites in groups of both SV vs MV and MV vs MG, we noticed that the secondary bile acid biosynthesis pathway of *Akkermansia* metabolites partially overlaps with the bile secretion pathway of colon metabolites. Interestingly, hydrocortisone, 18-β-glycyrrhetinic acid, berberine and the bile secretion pathway were all tightly associated with the inflammation response. For instance, hydrocortisone is well known for its anti-inflammation effect in treatment of many diseases, like autoimmune diseases and asthma, and especially, it improves the intestinal barrier functions [54, 55]; Glycyrrhetinic acid and berberine are the components of GQD and both were recognized as an anti-inflammation effect on intestinal mucosa [56, 57]. Besides, the intermediates or derivatives, e.g. lithocholic acid, deoxycholic acid and ursodeoxycholic acid, in the bile secretion pathway can promote the survival of *Akkermansia* in the intestine and inhibit the occurrence of colitis [58–61]. As such, we examined the permeability and the inflammation status in colon. As expected, GQD administration protected intestinal permeability and greatly alleviated the inflammation response induced by Meth withdrawal. Recently, the interplay among gut microbiome, gut inflammation and psychiatric disorders, such as anxiety and depression, have been recognized [53, 62–65]. Hence, we speculated that metabolites including hydrocortisone, glycyrrhetinic acid and berberine, as well as the bile secretion pathway are key metabolic linkers between *Akkermansia* and gut microenvironment that underlying therapeutic effects of GQD against Meth withdrawal anxiety.

## Conclusions

**Fig. 5:**
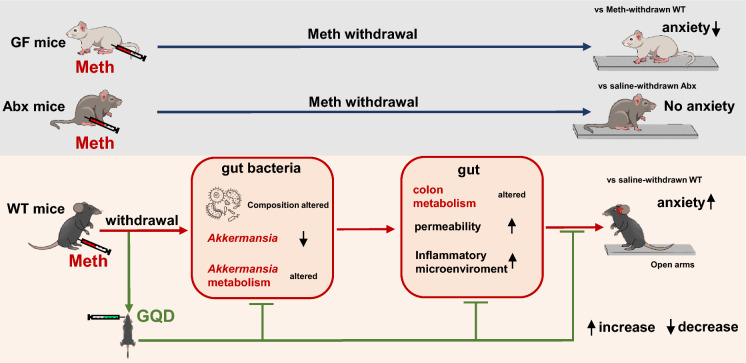
Schematic summary of this study. The Methamphetamine (Meth) withdrawal increases anxiety-like behaviors in wild-type (WT) mice, but is alleviated in germ-free (GF) mice, and does not influence that in antibiotics-treated (Abx) mice, indicating the essential role of gut bacteria in Meth withdrawal anxiety. Intragastric Gegen-Qinlian decoction (GQD) treatment during withdrawal period efficiently alleviates Meth withdrawal-induced anxiety in WT mice. The gut bacteria, especially *Akkermansia* and its metabolism, might be the original pharmacological targets of GQD, which then improved the gut metabolism, permeability and inflammatory microenvironments that were impaired in Meth-withdrawn mice

In conclusion, this study confirmed the role of gut microbiota in regulating the Meth withdrawal anxiety, and for the first time found that GQD can efficiently alleviate anxiety and gut bacteria in Meth-withdrawn mice. We targeted *Akkermansia* as the key bacteria by GQD to treat Meth withdrawal anxiety. By metabolomics profile of in vivo colon and in vitro* Akkermansia* culture, metabolites such as hydrocortisone, glycyrrhetinic acid and berberine, as well as the bile secretion pathway are key metabolic linkers between *Akkermansia* and gut microenvironment that underlying therapeutic effects of GQD against Meth withdrawal anxiety (Fig. 5). Our findings suggest that targeting gut bacteria, such as by GQD, might be a promising therapeutic strategy for the addiction and related withdrawal symptoms.

## Data Availability

All data generated or analyzed during this study are included in this published article [and its supplementary information files].
